# Humanization of fibroblast growth factor 1 single‐chain antibody and validation for its antitumorigenic efficacy in breast cancer and glioma cells

**DOI:** 10.1111/jcmm.13547

**Published:** 2018-03-24

**Authors:** Xiao‐Xiao He, Shuang Du, Shi‐Qian Gao, Jing‐Ying Chen, Ran‐Juan Cao, Zhen‐Kai Xing, Alia Rizvi Syeda Kazim, Hua‐Li Yu, Qing‐Chuan Zheng, Xiao‐Juan Zhu

**Affiliations:** ^1^ Key Laboratory of Molecular Epigenetics of Ministry of Education Institute of Genetics and Cytology Northeast Normal University Changchun China; ^2^ Department of Hand Surgery China‐Japan Union Hospital of Jilin University Changchun China; ^3^ Laboratory of Theoretical and Computational Chemistry Institute of Theoretical Chemistry Jilin University Changchun China

**Keywords:** antibody humanization, cancer treatment, fibroblast growth factor 1, monoclonal antibody, single‐chain variable fragment

## Abstract

Single‐chain variable fragment (scFv) antibodies are the smallest immunoglobulins with high antigen‐binding affinity. We have previously reported that fibroblast growth factor 1 played pivotal roles in cancer development and generated a mouse scFv (mscFv1C9) could effectively prohibit cancer cell proliferation in vitro and in vivo. Here, we further humanized this scFv (hscFv1C9) using a structure‐guided complementarity determining region grafting strategy. The purified hscFv1C9 maintained similar antigen‐binding affinity and specificity as mscFv1C9, and it was capable of inhibiting growth of different tumours in vitro and in vivo. These data strongly suggested that hscFv1C9 has antitumour potentials.

## 1 INTRODUCTION

Acidic fibroblast growth factor (fibroblast growth factor 1, FGF‐1), a member of the fibroblast growth factor superfamily, has multiple functions in DNA synthesis, cell division and differentiation, blood vessel diastole and hormone secretion.^1^ It was also critical for the development of different cancers.^2^, ^3^, ^4^ Thus, FGF‐1 has been proposed to be a cancer therapy target, and this approach has been validated using specific inhibitor of FGF receptor (FGFR).^5^, ^6^ Monoclonal antibody‐mediated immunotherapy is an accepted treatment modality by FDA and has shown great potential in clinical settings.^7^, ^8^ However, the feasibility of using monoclonal antibodies that specifically target FGFR1 is impeded by its poor uptake rate and high immunogenicity.^9^ Single‐chain variable fragment (scFv) is another option which maintains the antigen‐binding activity and is of a smaller size. Successful applications of scFv against FGFR have been already documented.^10^, ^11^ In our previous work, a mouse scFv antibody (hereafter: mscFv1C9) targeting FGF‐1 was generated.^12^ Expression of mscFv1C9 effectively arrested cancer cells at the G0/G1 transition and showed potential to treat breast cancer in vivo.^12^, ^13^ However, similar to most scFv, mscFv1C9 was generated from mouse cell lines, which will inevitably cause human antimouse antibody (HAMA) reactions.^14^ HAMA reactions caused strong allergic reactions and accelerated the clearance of scFv in vivo.^15^ Here, we humanized mscFv1C9 (called hscFv1C9) and validated its effects using in vitro and in vivo studies.

## 2 MATERIALS AND METHODS

NCBI/IGBLAST with default setting was used for templates selection. Interchain packing residues were identified according to http://people.cryst.bbk.ac.uk/~ubcg07s/. Unusual residues were identified using SeqTest on http://www.bioinf.org.uk/abs/. Protein was expressed in Rosetta *Escherichia coli* strain and then purified by affinity chromatography. For home‐made ELISA, 1 μg FGF‐1 or FGF‐2 was added to each well at 4°C overnight, followed by incubation with scFv for 1 hour at 37°C. After washing, HRP‐conjugated secondary antibody was used for protein‐binding detection. Cell proliferation was measured by CCK8 kit according to the manufacturer's protocol. U87 cells were injected subcutaneously into the left forelimb of female BALB/c nude mice. All mice were handled in accordance with the Guidelines of the Animal Care and Use Committee of Northeast Normal University. Mann‐Whitney *U* test was used for statistical analysis.

## 3 RESULTS AND DISCUSSION

### 3.1 Humanization of mscFv1C9

A structure‐guided complementarity determining region (CDR) grafting approach was employed to humanize mscFv1C9. The VH and VL amino acid sequences of mscFv1C9 were separately compared to human germline antibody sequences using NCBI/IGBlast. The top 8 matching human antibody sequences were retrieved for further analysis. Pairwise distance's analysis using MEGA3.1 showed IGHV3‐21*04, IGHV3‐48*03 and IGHV3‐23*03 were highly homologous to mscFv1C9‐VH, whereas IGKV1‐39*01 had the highest homology score to mscFv1C9‐VL (Table S1). Taking into consideration the length of each sequence, IGHV3‐48*03 and IGKV1‐39*01 were chosen as the templates for VH and VL chain, respectively. Next, CDRs in mscFv1C9‐VH and VL were defined using the Kabat system. The vernier residues (Table S2) and the interchain packing residues (Table S3) of mscFv1C9 were also identified according to previous reports. Lys106 of mscFv‐VL had an extremely low occurrence rate (0.542%) in the mouse antibody database. Thus, all these residues were maintained during humanization.

**Figure 1:**
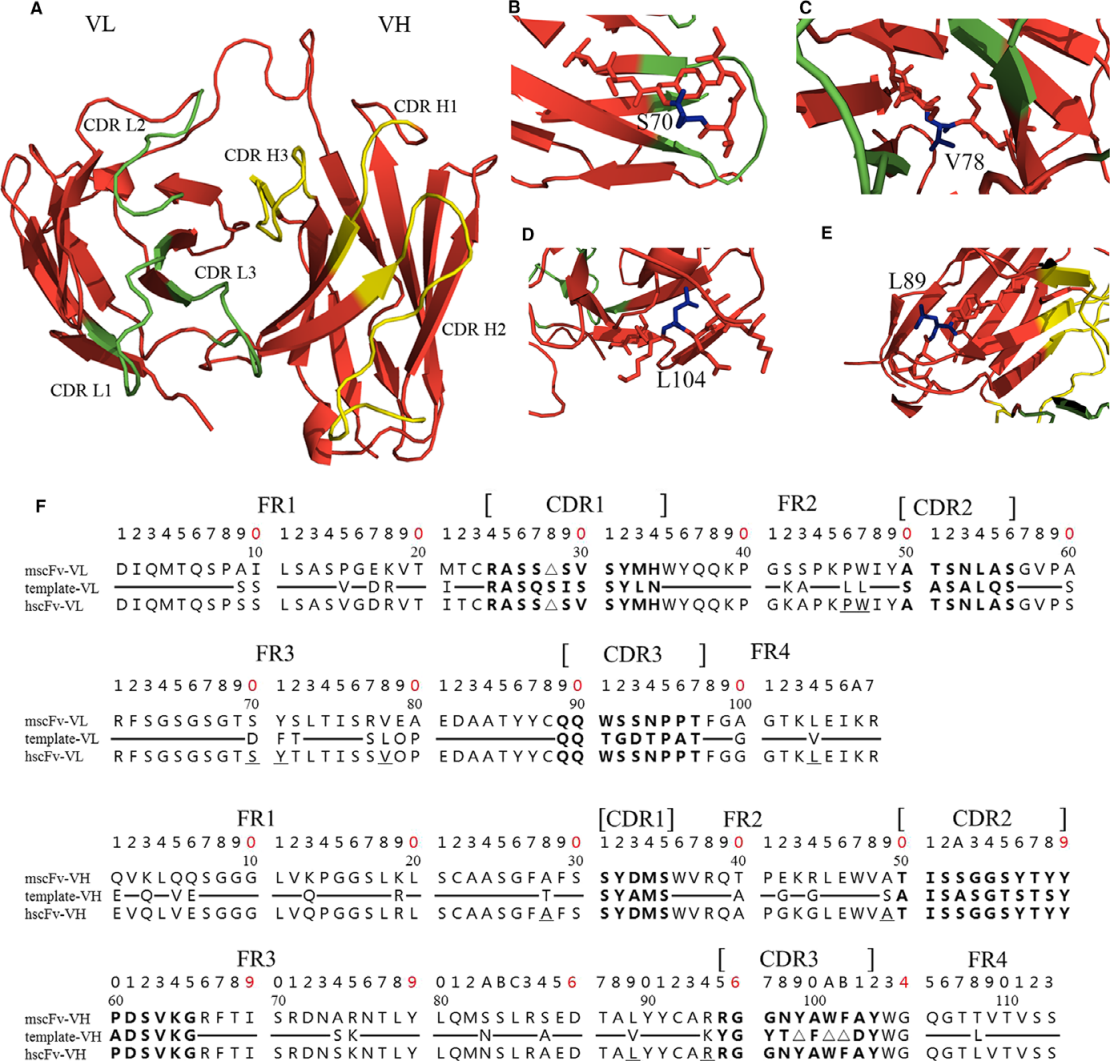
Humanization of mscFv1C9. (A) The 3D structure of mscFv1C9 was generated using PyMOL software. Complementarity determining regions (CDR)s in VL were highlighted in green, and CDRs in VH were highlighted in yellow. Ser70 (B), Val78 (C) and Leu104 (D) in VL and Leu89 (E) in VH were identified as essential for binding affinity, thus were back‐mutated in the following humanization process. (F) Numbering system of VL and VH used the Kabat numbering scheme. The CDRs of VL and VH are shown in Bold. Amino acid sequences that are different between mscFv and the human templates are listed in the figure, otherwise shown as ━. Redundant amino acid due to the length difference was represented by ▵. Back‐mutated residues were underlined

Next, the 3D structure of mscFv was created based on the protein structure of 2KH2 using the software Insight II. Consistent‐valence force field was used for energy minimization and molecular dynamics simulation. Biological plausibility of mscFv1C9 was checked using Profiles‐3D and Procheck. PyMOL software was used to visualize the predicted structure (Figure 1A) and to analyse the individual residues that were within 5 Å of CDRs and the residues were at the active sites for antigen binding. After a manual screening, Ser70, Val78 and Leu104 in mscFv1C9‐VL and Leu89 in mscFv1C9‐VH were maintained (Figure 1B‐E). Finally, 11 residues on human FR templates were back‐mutated to the corresponding mouse antibody residues, and the hscFv1C9 was generated (Figure 1F). Though 11 back mutations were not trivial compared to the total length of the hscFv1C9, Z evaluation and LakePharma antibody analyser showed hscFv1C9 had higher humanization score than mscFv1C9 (Table S4).

The hscFv1C9 was de novo synthesized and codon optimized for *E. coli* expression (Figure S1). The constructed hscFv1C9 sequence was subcloned into pET22b, which possesses an N‐terminal PelB signal peptide for protein secretion and a C‐terminal His‐tag for protein purification. A laboratory‐scale high‐density fermentation (5 L) was used to express hscFv1C9 with following conditions: 0.2 mmol/L IPTG and 6‐hour induction at 28°C (Figure S2A). The purity of the target protein was assessed by SDS‐page and Western blotting using anti‐His antibody (Figure S2B,C). A total of 400 mg hscFv1C9 was purified from 1 fermentation experiment.

### 3.2 hscFv1C9 inhibited cancer cell growth in vitro and in vivo

**Figure 2:**
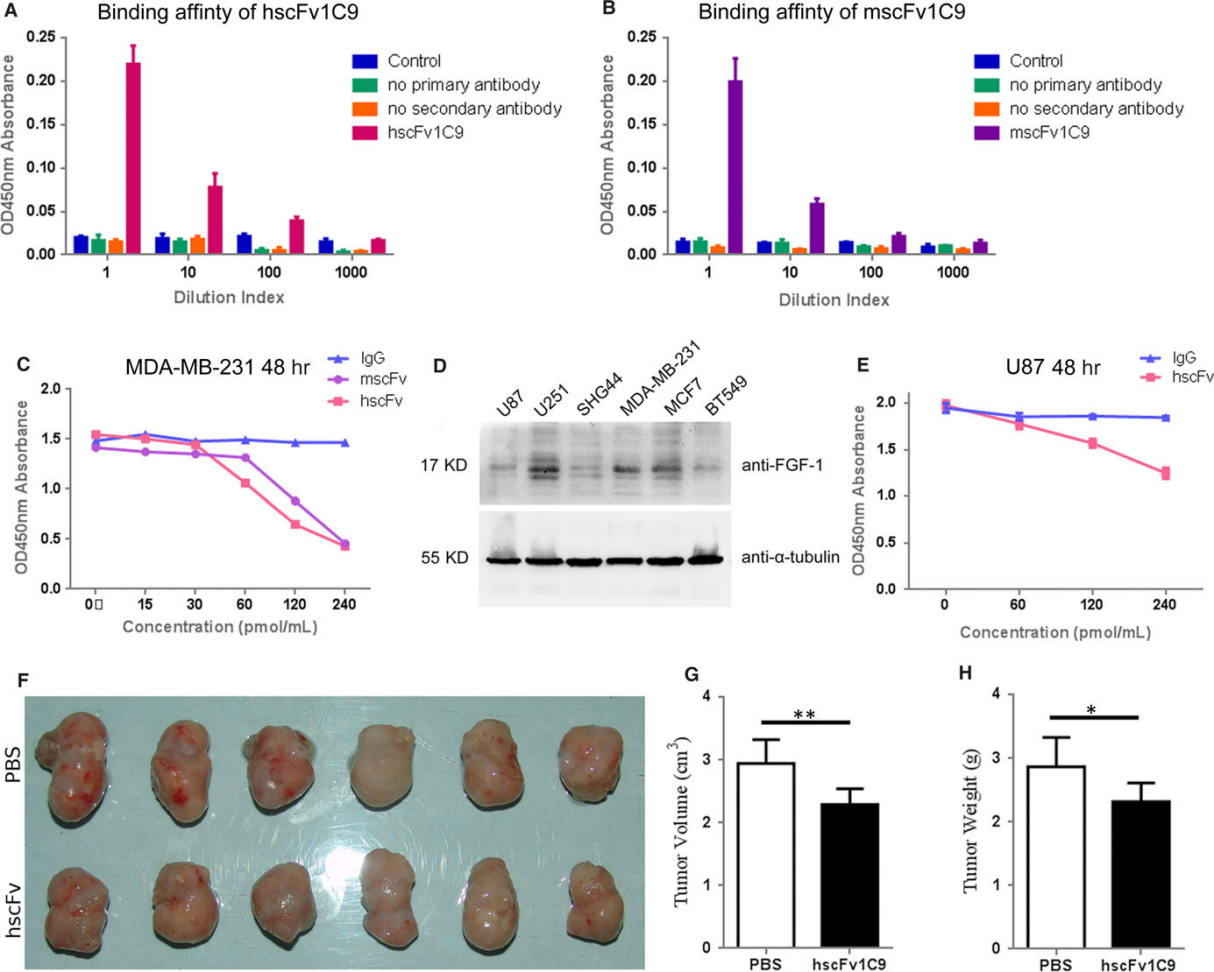
hscFv1C9 inhibited tumour cells growth in vitro and in vivo. Home‐made ELISA showed hscFv1C9 (A) and mscFv1C9 (B) have similar binding affinity to FGF‐1 at comparable dilution. A CCK‐8 assay was used to measure cell density after the indicated treatment. PBS was used in 0 pmol/mL group as the control of this assay. (C) Both hscFv1C9 and mscFv1C9 showed inhibitory effects on MDA‐MB‐231 cell proliferation in a dosage‐dependent manner. MDA‐MB‐231 cells were incubated with antibodies for 48 h and followed by CCK‐8 reaction for 180 min. (D) Western blotting analysis of FGF‐1 expression in glioma and breast cancer cell lines. (E) U87 cells were incubated with hscFv1C9 for 48 h and followed by CCK‐8 reaction for 120 min. Cell growth of U87 cells was prohibited by hscFv1C9. (F) Isolated tumours from nude mice. (G) and (H) Statistical analyses of tumours size and tumour weight from 2 groups. Mann‐Whitney U test was used for statistical analysis. Data were shown as Mean ± *SD*. ** indicated *P* value < .01; * indicated *P* value < .05

A home‐made ELISA was used to test the binding affinity of purified hscFv1C9 to FGF‐1 using mscFv1C9 as a positive control^12^ (Figure S2D,E). At each concentration, hscFv1C9 and mscFv1C9 had comparable binding affinity (Figure 2A,B). Similar to mscFv1C9,^12^ hscFv1C9 did not bind significantly to FGF2 (Figure S3A). The binding affinity between hscFv1C9 and FGF‐1 was greatly reduced when free FGF‐1 peptide was added to the ELISA reaction (Figure S3B).

Next, we tested the effects of hscFv1C9 in cancer cells. As we had previously tested mscFv1C9 in breast cancer cells,^12^, ^13^ MDA‐MB‐231 was firstly used in cell proliferation and cell cycle tests. Both hscFv1C9 and mscFv1C9 effectively inhibited cell proliferation at high concentrations (Figure 2C), and arrested cell cycles at G0/G1 (Figure S3C). Thus, the cellular effects of hscFv1C9 were similar to that of mscFv1C9.

We then tested hscFv1C9 in glioma cells to further investigate the antitumour effect of hscFv1C9. Glioma is a kind of infiltrating cancer that usually cannot be completely removed in surgery and was reported require FGF‐1 during gliomagenesis.^16^, ^17^ Safe and effective drugs are urgently needed to treat these kinds of tumours. Therefore, it is valuable to generate new compound that has therapeutic potential against glioma. Western blotting analysis showed U87 glioma cells had relatively lower endogenous FGF‐1 expression (Figure 2D). As expected, hscFv1C9 inhibited U87 cells proliferation in a dosage‐dependent manner (Figure 2E). Then, we generated glioma xenograft mice model using U87 cells. Freshly collected cells were subcutaneously injected in the left forelimbs of 12 nude mice. The hscFv1C9 treatment was started at 10 days after initial injection when tumour formation was seen in all nude mice. The treated mice received 4 times injections of hscFv1C9 (25 mg/kg, injection volume ≤ 25 μL) at 4‐day intervals at the tumour site (Figure S4A), whereas control mice received 25 μL of PBS. Four days after the last treatment (26th day), all mice were killed, and tumours were isolated (Figure S4B). Tumours in the treatment group were visibly smaller than that of control group (Figure 2F). Statistical analyses confirmed that tumours were significantly smaller and lighter in the treated mice (Figure 2G,H). This provided evidence that hscFv1C9 may have a strong inhibiting effect on gliomagenesis.

In conclusion, we successfully humanized a FGF‐1 single‐chain antibody and examined its cellular effects in breast cancer and glioma cells in a proof‐of‐principle study. More importantly, we tested the antitumorigenic efficacy of hscFv1C9 using a xenograft glioma mice model. Revealing the working mechanism of hscFv1C9 is needed to promote its medical possibility. We had previously shown that the intracellular expressed mscFv1C9 arrested cell cycles via inhibiting the FGF‐1 intracrine pathway.^12^, ^13^ However, FGF‐1 works in both intracrine and paracrine pathways. Consequently, when the purified hscFv1C9 was administered in a different method, the antitumour effect may be caused by a different working mechanism, which required further examination. Nevertheless, this study demonstrated that a novel humanized scFv against FGF‐1 has antitumour potentials in cancer treatment.

## CONFLICT OF INTEREST

The authors have no potential conflict of interests to disclose.

## AUTHORS CONTRIBUTIONS:

X.H., H.Y., Q.Z. and X.Z. designed the study and wrote the manuscript. X.H., S.D., S.G., J.C., R.C., Z.X. and A.R. performed the experiments and analysed the data.
