# Supplementary material for: Humanization of fibroblast growth factor 1 single‐chain antibody and validation for its antitumorigenic efficacy in breast cancer and glioma cells
Source: J Cell Mol Med. 2018 Mar 24;22(6):3259–63. doi: 10.1111/jcmm.13547 (PMC5980129; doi:10.1111/jcmm.13547)
Supplement: Supplementary file 1 [file JCMM-22-3259-s001.docx]

Supplemental Materials

Table S1 Pairwise Distances analysis of mscFv1C9-VL and VH to the corresponding human antibody templates

| VH templates | Score |  |  |  |  |  |  |  |
| --- | --- | --- | --- | --- | --- | --- | --- | --- |
| IGHV3-48*03 | 0.2603 |  |  |  |  |  |  |  |
| IGHV3-21*04 | 0.2469 | 0.0757 |  |  |  |  |  |  |
| IGHV3-23*03 | 0.2469 | 0.1455 | 0.1455 |  |  |  |  |  |
| IGHV3-NL1*01 | 0.2739 | 0.1455 | 0.1455 | 0.0426 |  |  |  |  |
| IGHV3-11*04 | 0.2603 | 0.087 | 0.0757 | 0.1455 | 0.1576 |  |  |  |
| IGHV3-11*04_CAR62555.1 | 0.4372 | 0.3302 | 0.3017 | 0.3747 | 0.3597 | 0.2469 |  |  |
| IGHV3-23*05 | 0.2603 | 0.1335 | 0.1335 | 0.0211 | 0.0645 | 0.1335 | 0.3597 |  |
| IGHV3-23*04_CAR63034.1 | 0.2877 | 0.2076 | 0.2469 | 0.1576 | 0.1823 | 0.2603 | 0.4868 | 0.1699 |
| VL templates |  |  |  |  |  |  |  |  |
| IGKV1D-39*01 | 0.3889 |  |  |  |  |  |  |  |
| IGKV1-NL1*01 | 0.4223 | 0.0931 |  |  |  |  |  |  |
| IGKV1-27*01 | 0.5486 | 0.2371 | 0.2513 |  |  |  |  |  |
| IGKV1-39*01_CBZ39890.1 | 0.3889 | 0 | 0.0931 | 0.2371 |  |  |  |  |
| IGKV1-39*01_CBZ39887.1 | 0.3889 | 0 | 0.0931 | 0.2371 | 0 |  |  |  |
| IGKV1-39*02 | 0.4745 | 0.156 | 0.1823 | 0.2371 | 0.156 | 0.156 |  |  |
| IGKV1-9*01 | 0.4055 | 0.0931 | 0.1178 | 0.2094 | 0.0931 | 0.0931 | 0.081 |  |
| IGKV1-17*01 | 0.4925 | 0.1691 | 0.1957 | 0.2803 | 0.1691 | 0.1691 | 0.1823 | 0.1178 |

Table S2 Venier residues of mscFv1C9-VL and VH

| VL | | VH | |
| --- | --- | --- | --- |
| 2  4  35-36  46-L49  64  66  68-69  71  98 | I  M  WY  PWIY  G  G  GT  Y  F | 2  27-30  47-49  67  69  71  73  78  93-94  103 | V  FAFS  WVA  F  I  R  N  L  AR  W |

Venier residues were analyzed according to Foote J, Winter G. Antibody framework residues affecting the conformation of the hypervariable loops. J Mol Biol. 1992; 224: 487-99.

Table S3 The interchain packing residues of mscFv1C9

| VL | | VH | |
| --- | --- | --- | --- |
| **34**  36  38  44  46  87  **89**  **91**  98 | **H**  Y  Q  P  P  Y  **Q**  **W**  F | **35**  37  39  45  47  91  93  **95**  103 | **S**  V  Q  L  W  Y  A  **R**  W |

The interchain packing resisdues were identified according to <http://people.cryst.bbk.ac.uk/~ubcg07s/>. Letters in **Bold** are in CDRs.

Table S4 Comparison of humanness of hscFv1C9 and mscFv1C9

| Method | hscFv1C9-VH | hscFv1C9-VL | mscFv1C9--VH | mscFv1C9-VL |
| --- | --- | --- | --- | --- |
| Z evaluation | 1.253 | 0.287 | 0.482 | -0.965 |
| T20 score | 89.88 | 63.13 | 78.20 | 59.69 |

Z evaluation was used an online server (<http://www.bioinf.org.uk/abs/shab/>).

T20 score were used online server (<https://dm.lakepharma.com/bioinformatics/>) with framework only option.

In both systems, higher score means more humanness.


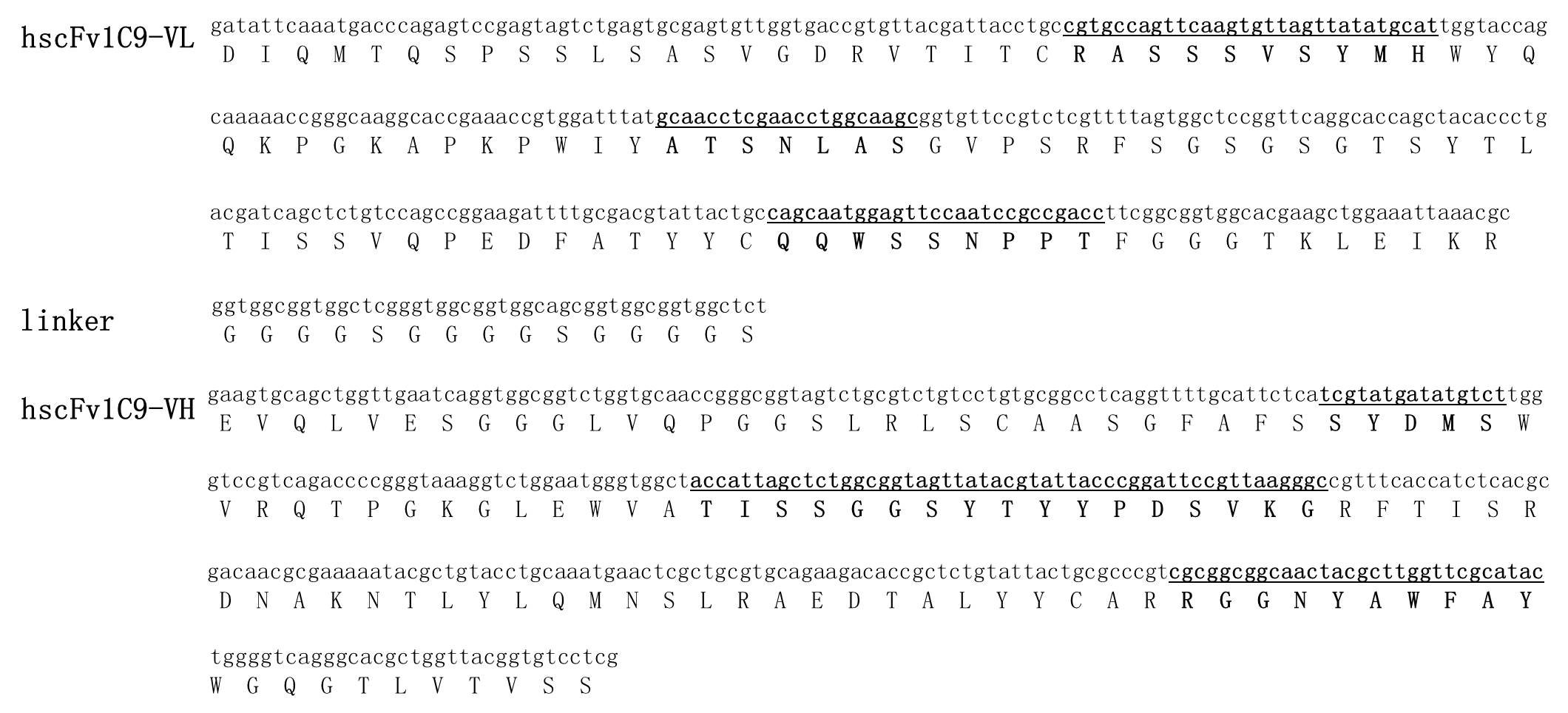


Figure S1 Nucleotide sequences of hscFv1C9

Nucleotide sequences of hscFv1C9 were *de novo* synthesized and codon-optimized for *E.coli* expression system.

**
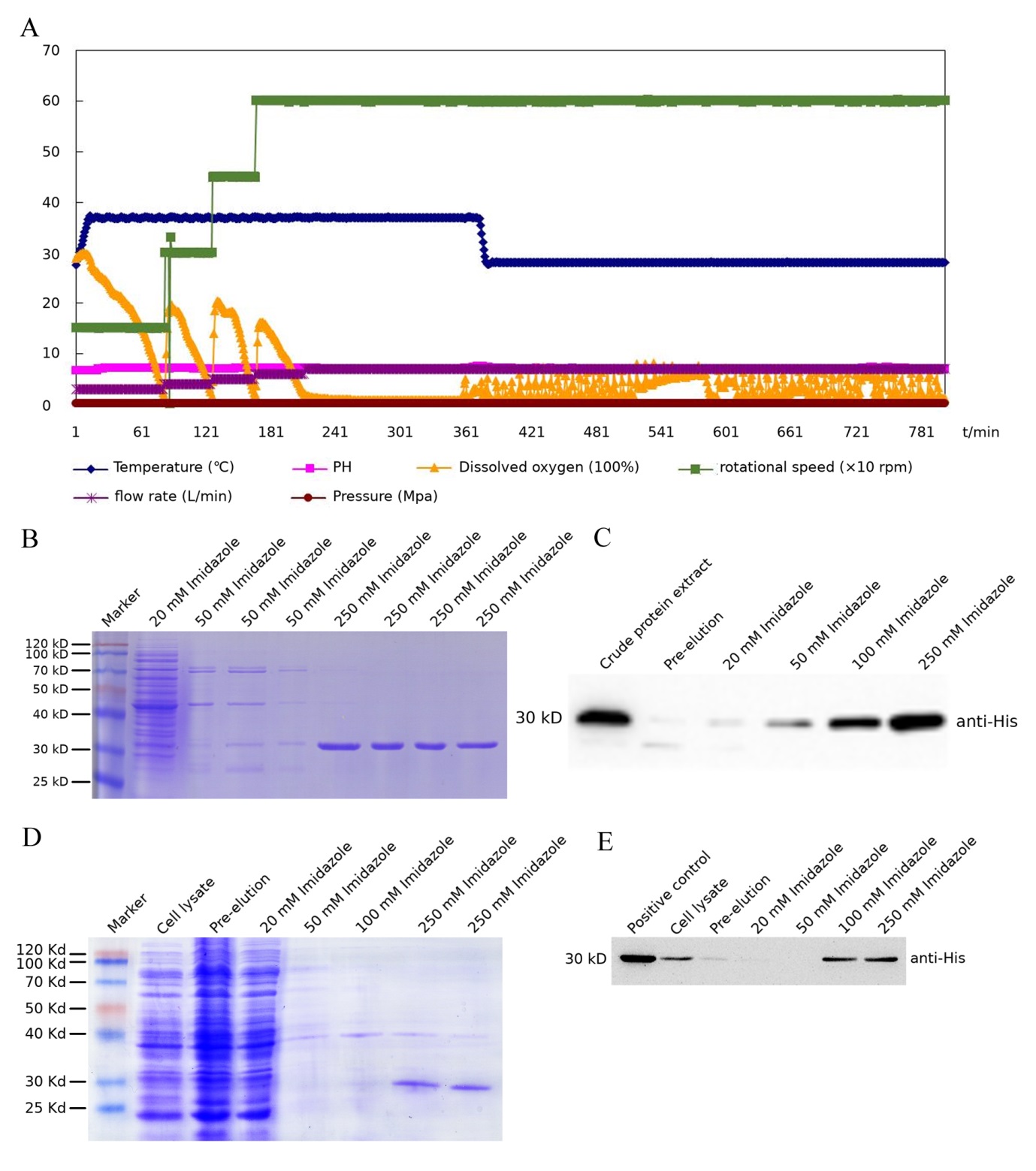
**

Figure S2 Production of hscFv1C9 and mscFv1C9

(A) The optimal conditions for soluble hscFv1C9 expression were 0.2mM IPTG and 28 °C for 6 h. Lab-scale fermentation used the same expression conditions with extra control of PH and oxygen input. Parameters of the fermentation were monitored during the whole process. (B and C) SDS-page and western blotting confirmation of purified hscFv1C9. (D and E) SDS-page and western blotting confirmation of purified mscFv1C9. The positive control band in western blot was the purified hscFv1C9.


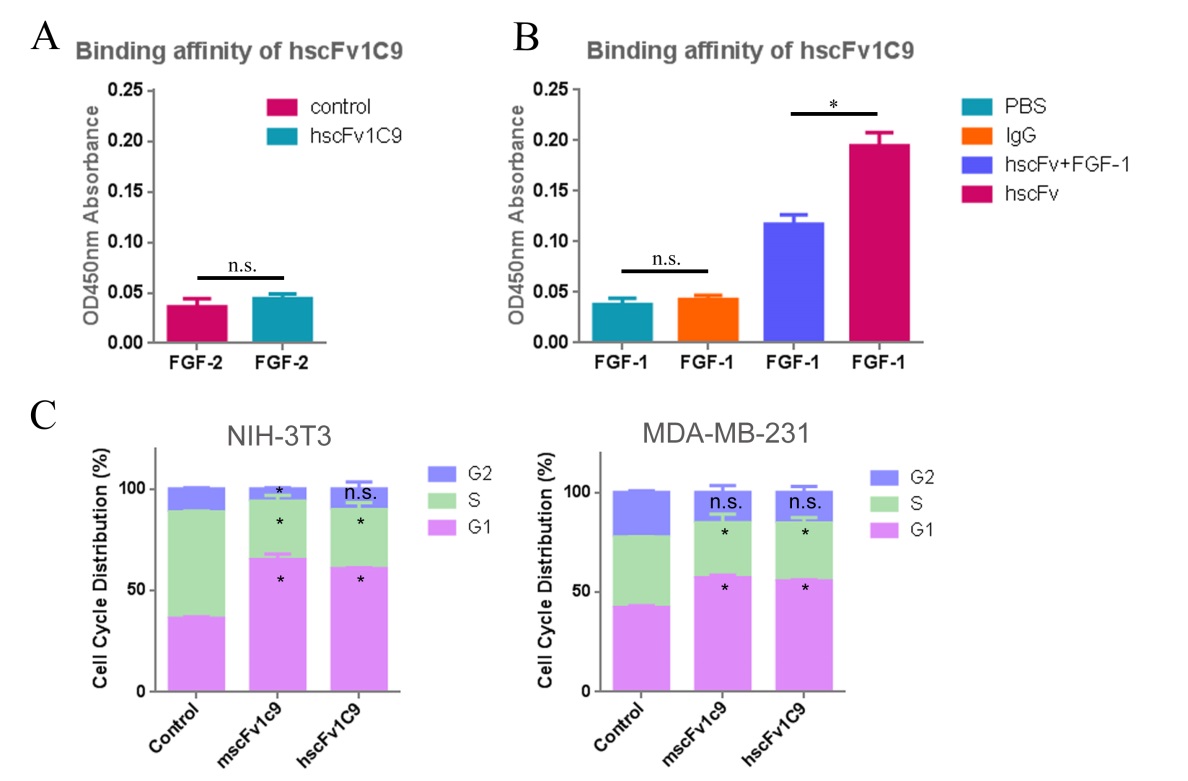


Figure S3 Cellular effects of hscFv1C9

(A) hscFv1C9 did not significantly bind to FGF-2 in ELISA. Undiluted hscFv1C9 was used. (B) IgG and PBS had similar binding affinity to FGF-1. Competitive elusion with free FGF-1 peptide (1μg) significantly reduced binding affinity between hscFv1C9 and coated FGF-1. (C) Both mscFv1C9 and hscFv1C9 arrested cell cycles at G0/G1 for NIH-3T3 and MDA-Mb-231 cells. Mann-Whitney U test was used for statistical analysis. Data was shown as Mean±SD. * indicated P value < 0.05


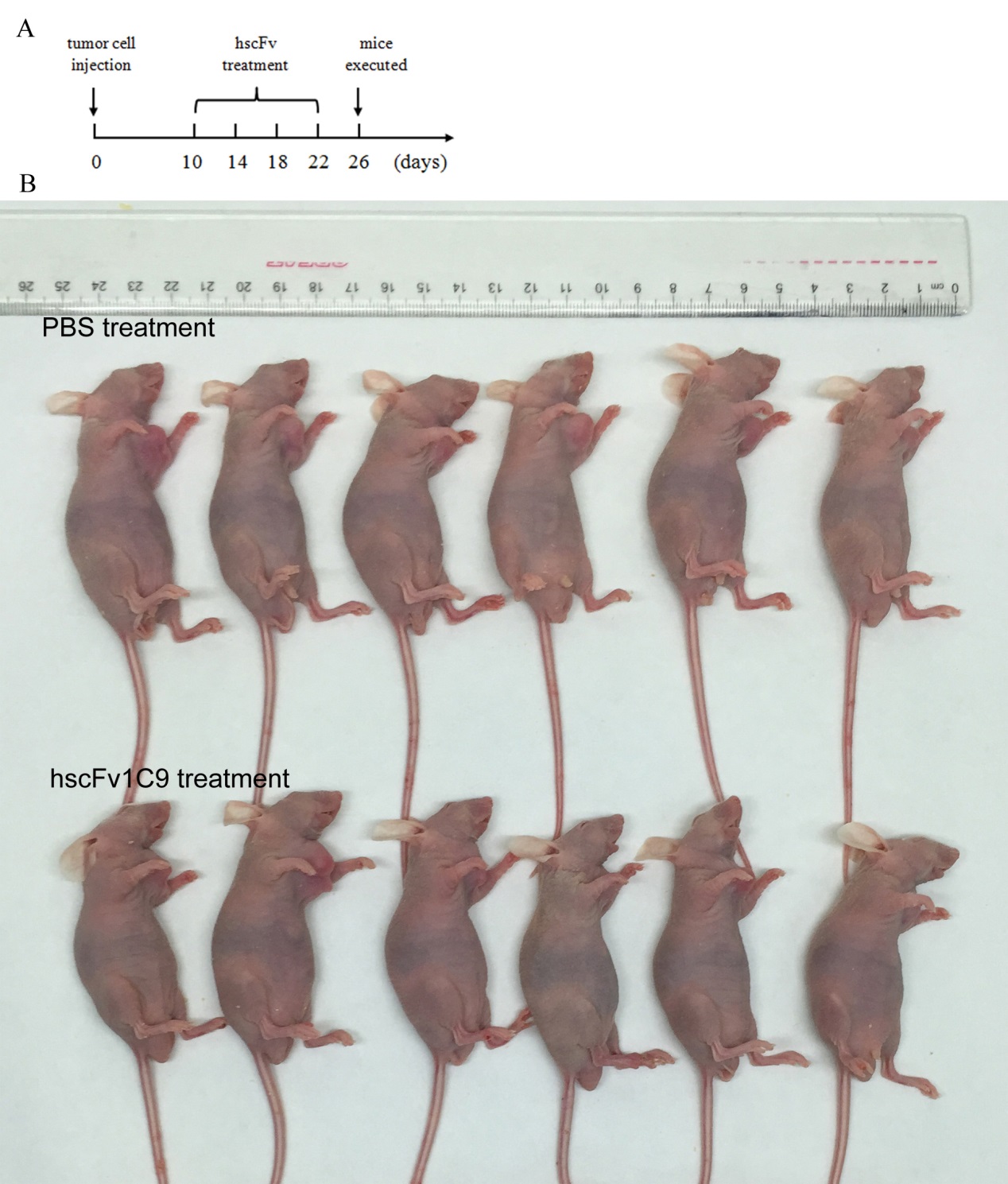


Figure S4 *In vivo* tumor formation in nude mice

(A) Outline of xenograft mice model establishment and the subsequent hscFv1C9 treatment. (B)Tumor formation at the injection site was seen in all mice from 10^th^ days post tumor implantation. Picture was taken on the 26^th^ day, before all mice were sacrificed for tumor isolation.

**Supplemental Materials and Methods**

**Software analysis**

For templates selection, sequences searched in NCBI/IGBLAST server (<https://www.ncbi.nlm.nih.gov/igblast/>) using default setting. Interchain packing residues were identified according to <http://people.cryst.bbk.ac.uk/~ubcg07s/> . Unusual residues were identified using SeqTest on <http://www.bioinf.org.uk/abs/>.

**Protein expression and purification**

The construct was transformed into Rosetta *E. coli* strain. Individual colonies were picked and grown in 5 mL LB media at 37°C overnight in the presence of 50 μg/mL ampicillin and 34 μg/mL chloramphenicol. For laboratory scale fermentation, all *E. coli* culture was inoculated to 5 L culture medium (10 g/L Glucose, 11.5 g/L yeast powder, 19.5 g/L peptone, 4 g/L (NH_4_)_2_SO_4_, 18 g/L K_2_HPO_4_, 1.2g /L MgSO_4_, 1 mL/L trace element solution). Protein expression was induced by 0.2 mM IPTG for 6h at 28°C. Then cells were harvested and broken by high pressure. The supernatant was filtered through a 0.22 μm filter, and concentrated by using ultrafiltration tube (Millipore, 10KD, UFC901008). The concentrated protein was loaded into a nickel resin column. Then the protein was washed by 20mM imidazole and 50mM imidazole to wash the unspecific bindings. Finally, the protein was eluted by 100mM imidazole and then 250mM imidazole. The purified protein was concentrated by using ultrafiltration tube and then dialysis with PBS at 4°C. The purified protein was stored at -80°C.

**Cells and cell culture**

Glioma cells U251, U87MG, SHG44, breast cancer cells MCF7, MDA-MB-231 and fibroblast cells NIH-3T3 were all originally obtained from ATCC (the American Type Culture Collection) and cultured according to standard procedure.

**ELISA**

For home-made ELISA, 100 μL FGF-1 or FGF-2 (10μg/mL) was added to 96-well microtiter plates at 4°C overnight. The coated-plates were washed four times with PBST and were blocked using 2% BSA for 1h at 37°C. Indicated hscFv1C9 or mscFv1C9 was added to the coated-plates for 1h at 37°C. For competitive elusion, 100 μL (1μg) of FGF-1 was added to the well following addition of hscFv1C9. Anti-His antibody was added to each well and incubated for 1h at 37°C (ZSGB-BIO, 1:1000). Then washed four times with PBST. The HRP-conjugated second antibody (rabbit anti-mouse IgG, 1:15000) was added to each well and incubated for 1h at 37°C. Washed as before and added 200 μL TMB for 30 min at room temperature in dark. The reaction was stopped by addition 50 μL of 2M H_2_SO_4_ and the optical absorbance of each well was measured at 450 nm. Control mouse IgG was purchased from Proteintech (B900620).

**Immunoblotting**

Western blotting was carried out according to Bio-Rad’s General Protocol (BIO-RAD Bulletin 6376 Rev A). Antibody against His tag was purchased from ZSGB-BIO (1:500). The secondary antibody was purchased from ZSGB-BIO (1:5000).

**Cell proliferation analysis**

Cell proliferation was measured by CCK8 kit (Dojindo Molecular Technologies, CK04). 5 x 10^3^ cells were seeded in each well of a 96-well plate overnight. Then scFvs or mouse IgG were added to each well at the indicated concentration for certain periods. After incubation, 10 µl of CCK-8 solution was added to each well. The reactions were incubated for 1-3 hours at 37°C. Cell viability was determined by measuring the absorbance at 450 nm. Control mouse IgG was freshly prepared from healthy mice serum using Pierce^TM^ Protein A/G magnetic beads (Thermo, 88802) with manufacturer’s protocol. The purified IgG was sterilized by 0.45 µm Millex-HV filter (Merck SLHV033RS).

**Cell cycle analysis**

Cells were starved for 20 h, and then treated by scFv for 24 h. All cells were resuspend in 300 μL PBS and fixed in 700 μL 70% ethanol on ice for 15 min. Then cells were centrifuged at 4000g for 5min. Cells were washed twice with PBS, and then were treated with 20 μL RNase (1mg/mL) for 30min at 37°C. Then, centrifuge the cells again to remove any supernatant. Cells were resuspended in 200 μL PBS and stained with 200 μL PI for 10min at room temperature. The cell cycle analysis was performed using a ﬂow cytometer.

**Xenograft tumor mice model**

Female BALB/c nude mice were purchased from Wei Tong Li Hua Company (Beijing). All mice were handled in accordance with the Guidelines of the Animal Care and Use Committee of Northeast Normal University. All cages, bedding and water were autoclaved before use. Mice were anesthetized by pelltobarbitalum natricum (210µg/20g) and placed in a stereotactic frame with a mouse adaptor. 5 x 10^6^ U87MG cells were injected into the left forelimb subcutaneously. After 10 days, the average size of the tumor was 100mm^3^. Then the mice implanted with U87MG cells were randomly grouped to PBS control and 25mg/kg hscFv1C9 treatment groups. The control groups received 25 μL PBS and treatment groups received 25mg/kg hscFv1C9 by injected into the tumors sites every four days for 4 times. After 26 days, mice were euthanized and tumors were isolated.

**Statistical analysis**

All statistical analyses were performed using the GraphPad6 software. Mann-Whitney U test was chosen for comparing the means of two groups. ** indicates P value < 0.01, * indicates P value < 0.05, n.s. indicates no significant difference.
